# Editorial: Current Trends in Deep Learning for Movement Analysis and Prosthesis Control

**DOI:** 10.3389/fnins.2022.889202

**Published:** 2022-04-25

**Authors:** Ali H. Al-Timemy, Claudio Castellini, Javier Escudero, Rami Khushaba, Silvia Muceli

**Affiliations:** ^1^Biomedical Engineering Department, Al-Khwarizmi College of Engineering, University of Baghdad, Baghdad, Iraq; ^2^Department of Artificial Intelligence in Biomedical Engineering (AIBE), Friedrich-Alexander Universität Erlangen-Nürnberg, Erlangen, Germany; ^3^DLR - German Aerospace Center, Institute of Robotics and Mechatronics, Cologne, Germany; ^4^School of Engineering, Institute for Digital Communications, The University of Edinburgh, Edinburgh, United Kingdom; ^5^Australian Center for Field Robotics, University of Sydney, Chippendale, NSW, Australia; ^6^Department of Electrical Engineering, Chalmers University of Technology, Gothenburg, Sweden

**Keywords:** deep learning, deep neural network, myoelectric control, neuroprosthetics, kinematics

The control of assistive devices such as neuroprostheses and exoskeletons via bio-signals [electromyography (EMG), electrical impedance tomography (EIT), peripheral nerves signals (PNSs), etc.] is a promising research avenue in the field of function restoration for individuals with impaired movement (Phinyomark and Scheme, 2018). Movement analysis based on similar signals and kinematics plays an important role to improve the diagnosis and monitoring the progression of movement disorders.

Deep learning (DL) methods such as Convolutional Neural Networks (CNNs) and Recurrent Neural Networks (RNNs), can play an important role as an assistive diagnostic tool or in the control of devices, due to their potential to achieve high performance in classification, regression, and prediction tasks. However, the widespread and practical implementation is still limited and needs to be further investigated.

This collection includes 6 articles whereof 5 explored DL methods for prosthesis control based on EMG (Campbell et al.; Ghaderi et al.; Olsson et al.), EIT (Leins et al.), or PNSs (Luu et al.), and one (Lecomte et al.) explored DL for kinematics analysis.

Ghaderi et al. proposed a feature extraction method based on kernel density estimation and evaluated it on single and concurrent classification tasks for the purpose of prosthesis control. For single tasks, a correlation-based feature selection was used, and the features were then classified using linear discriminant analysis (LDA). A classification accuracy of 98.99 ± 1.36 and 92.25 ± 9.48% was obtained for able-bodied and amputee subjects, respectively. The XGBoost classifier was also used for the classification of two movements simultaneously performed. The overall accuracy was 99.71 ± 0.08 and 97.85 ± 0.10% for the XGBoost and LDA classifiers, respectively.

Leins et al. investigated the use of EIT for the recognition of 12 hand gestures. They analyzed a dataset collected with a 16-electrode wrist band. Stronger inter-session and inter-user variance compared to the expected in-class variance, was observed by visualizing the EIT data with *t*-distributed stochastic neighbor embedding (*t*-SNE). A new machine learning architectures based on CNN was proposed to improve the classification accuracy. The cross-session classification increased from 19.55 to 30.45% with the new proposed CNN architectures. Finally, based on a fundamental data analysis, three calibration methods were developed, which yielded an increase of the cross-session classification accuracy to 39.01, 55.37, and 56.34%.

An asset of DL methods is that they can learn gesture classification tasks directly from the raw data (Côté-Allard et al., 2019) through multiple network layers. However, their computational cost may jeopardize real-time performance. Luu et al. explored different approaches to optimize the motor intent decoding from an amputee PNSs for real-time applications. They showed that the addition of a feature extraction step allowed networks with 4–5 layers to obtain similar performances as deep networks with 26 layers. Also, a RNN was able to regress the trajectory of the 5 fingers during 9 gestures more accurately than classic machine learning techniques (MLTs), confirming the DL advantage in handling large datasets. However, if the regression task was preceded by a classification step to identify the intended gesture, MLTs performed similarly to the DL ones, despite their simpler implementation. Luu et al. study underlies the importance of implementing motor decoding schemes efficiently to obtain real-time performance and maintain high accuracy.

On the same page, Olsson et al. employed Long Short-Term Memory (LSTM) networks to automatically extract prosthetic control commands from the raw intramuscular EMG signal. Three variations of the LSTM paradigm were compared in an experiment involving 14 able-bodied participants engaged in a set of repetitive tasks. Finger and wrist motion and forces were predicted, and two out of three of the LSTM methods outperformed a baseline linear regression method. The authors also go to some length to keep their systems manageable from the computational point of view. This is very agreeable, since in prosthetics, one always needs to take into account the wearability and energy consumption of the control system.

Campbell et al. explored a solution toward one of the main limitations in the development of DL models for biomedical signal processing applications: the fact that DL typically requires large training datasets but acquiring data from volunteers is difficult. Campbell et al. extended an adaptive domain adversarial neural network (ADANN) to a cross-subject framework. This enabled them to train the model with little data from the end-user. The proposed ADANN was evaluated thoroughly, comparing it with state-of-the-art hand-crafted feature sets, a CNN, and canonical correlation analysis as an alternative to adapt feature spaces across subjects. The results demonstrated that the cross-subject ADANN framework achieved high accuracy for both intact-limb and amputee subjects with just a single repetition of each movement class with a reasonable computational cost.

Finally, Lecomte et al. observed that existing solutions for marker-based animal movement analyses are expensive, difficult to manipulate, time-consuming, disruptive to the animal, and prone to human errors. The DeepLabCut^TM^ (DLC) open-access library was explored for vision-based movement analysis, without reflective markers-based systems. Four intact adult cats performed tied- and split-belt (same and different speeds) locomotion at left-right speed differences on a treadmill. Step and stride lengths, the horizontal distance of the toe from the hip at contact and liftoff, as well as hindlimb joint angles showed a significant agreement (above 90%) between the mean values of the variables obtained from DLC and a commercial software typically used in cat locomotor studies. The study demonstrated that DLC offered a potential for faster, more accurate, reduced variability and more repeatable outcomes than existing solutions, while remaining easily deployable, flexible, and low cost.

This Research Topic collected articles with the latest advances of DL methods in the field of movement analysis and prosthetic control. DL methods are known to require considerable computation resources and large datasets for training. However, in assistive and rehabilitation robotics, real-time performance is paramount. Also, obtaining large datasets from human participants suffering from musculoskeletal conditions or amputations can be challenging. Our article collection includes some efforts toward improving the efficiency of the motor decoding schemes and enabling DL network training with reduced datasets. These studies illustrate some of the future directions in the field.

## Author Contributions

All authors listed have made a substantial, direct, and intellectual contribution to the work and approved it for publication.

## Conflict of Interest

The authors declare that the research was conducted in the absence of any commercial or financial relationships that could be construed as a potential conflict of interest.

## Publisher's Note

All claims expressed in this article are solely those of the authors and do not necessarily represent those of their affiliated organizations, or those of the publisher, the editors and the reviewers. Any product that may be evaluated in this article, or claim that may be made by its manufacturer, is not guaranteed or endorsed by the publisher.
